# Open Access *Bacillus cereus* Cocktail Secondary Growth Model for the Food Industry

**DOI:** 10.3390/foods13213382

**Published:** 2024-10-24

**Authors:** Xiaoyang Tang, Dingwu Zhang, Pradeep K. Malakar

**Affiliations:** 1 Central Research Institute, Masterkong Holding, Shanghai 201103, China; xytang_vilin@163.com (X.T.);; 2 Shanghai Kangshi Food Science and Technology Co., Ltd., Shanghai 201103, China; 3 College of Food Science and Technology, Shanghai Ocean University, Shanghai 201306, China; 4 International Research Center for Food and Health, Shanghai Ocean University, 999# Hu Cheng Huan Road, Shanghai 201306, China

**Keywords:** *Bacillus cereus*, non-linear model, food industry

## Abstract

A cost-effective algorithm is presented, using a virtual dataset of growth rates from a cocktail of *Bacillus cereus* strains, for developing an open access, extended-range secondary growth model. Extended-range growth models can span the range of processing conditions typically used in food manufacturing and are therefore more relevant for industry. The open access extended-range secondary growth model for a cocktail of *B. cereus* strains was created using publicly available data, and the methodology can be adapted for modelling growth of other pathogens. An extended-range model can help manage *B. cereus* hazards in novel food categories with non-traditional formulations as estimations of *B. cereus* risks in these foods become more precise. This open access model, however, needs to be validated using data from *B. cereus* strain cocktails isolated from production facilities. Once validated, these independent factor models are valuable tools, in a pathogen decision support platform, which are tuned to local production environments. Such a platform can address the needs of current and future food product portfolios, effectively mitigating risks associated with *B. cereus* and other relevant pathogens.

## 1. Introduction

The Gram-positive bacterium, *Bacillus cereus*, is a facultative anaerobe capable of forming spores. It has been linked to foodborne illnesses resulting from the consumption of various contaminated food products [1]. Under favourable conditions, *B. cereus* can proliferate and produce both diarrheal and emetic toxins [2]. A significant increase in *B. cereus* populations to levels of 10^5^ CFU/g and above suggests the presence of toxins and the potential onset of disease [3].

The current industry approach to ensuring food safety focuses on measuring the *B. cereus* population per gram of product, rather than directly detecting the presence of toxins [4]. This is because once toxins are detected, pinpointing the reasons for their occurrence downstream becomes more challenging. Additionally, the exact population threshold at which toxins are produced is still uncertain [5]. Consequently, the industry has established acceptable thresholds for *B. cereus* at less than 100 CFU/g for infant milk products and less than 1000 CFU/g for food products intended for the general population.

Predictive microbiology models summarize the growth of cocktails of strains of *B. cereus*, inclusive of psychotropic and mesophilic strains, in broth as functions of temperature, pH, water activity (a_w_), and/or salt concentration (%NaCl). These models are available in the public domain (COMBASE) (Baranyi and Tamplin, 2004) and have proven useful for estimating risks within the domain of validity of these models [6]. The domain of validity of these models includes pH ranges from 4.9 to 7.4, temperature ranges from 5 to 34°C, and water activities from 0.94 to 1. Although these ranges of environmental conditions cater to most of the processing conditions encountered in food manufacturing, it would be useful to extend the range of validity of these models to accommodate more food production.

An addition to these models, using the latest taxonomic re-evaluation of the *B. cereus* group, is the study by Carlin et al. (2013) [7]. This re-evaluation involved a comprehensive analysis of the 16S rRNA gene sequences, as well as the use of whole-genome sequencing and phylogenomic analyses, to better understand the relationships among strains within the *B. cereus* group. The study demonstrates that individual *B. cereus* strains, which produce toxins affecting human health, can grow at temperatures between 1.4 and 55 °C and are also capable of growing at pH 4.5. These extended growth ranges may be relevant for assessing the safety of minimally processed foods. Of particular relevance to the food industry is the production of high-moisture cheeses, such as mozzarella, feta, and queso blanco, which have a final pH around 5.5 and rely on low storage temperatures as the primary food safety control option [8,9].

This manuscript aims to develop a procedure for combining publicly available *B. cereus* information and expressing the combined information in the form of an independent-factor, predictive microbiology *B. cereus* model. This open access model could be adapted further for the food industry. To achieve this goal, the procedure will involve the collection and analysis of relevant data from existing predictive microbiology models and research studies, as well as the integration of new findings from the taxonomic re-evaluation of the *B. cereus* group. The open access model will be designed to account for the extended growth ranges of *B. cereus* strains, thus providing a more comprehensive understanding of their behaviour in various food products.

However, we caution that the model we present is not a generalized model of *B. cereus* growth. The parameters of this particular model depend on the cocktail of *B. cereus* strains used. A generalized model of *B. cereus* growth may be possible but may need many more parameters.

Ultimately, this open access predictive microbiology model will serve as a valuable tool for food manufacturers, enabling them to better assess the safety of their products and implement more effective food safety control measures. 

## 2. Materials and Methods

Note that the data used in developing this approach are entirely derived from the public domain. No additional data were generated in a laboratory.

A source of data we used for designing this method was the COMBASE platform “https://browser.combase.cc/ (accessed on 15 October 2024)”. COMBASE is currently the most comprehensive information source for *B. cereus* growth and inactivation. The consortium quantifies the growth of *B. cereus* using ordinary differential equations (ODE). The ODE represents the bacterial cell population (x), the population growth rate (*μ*_max_), the growth medium environment (E), and Michaelis–Menten kinetics (q) [10].

(1) $$\frac{dx\left(t\right)}{dt}\frac{1}{x}=\frac{q\left(t\right)}{1+q\left(t\right)}\mu \left(E\left(t\right)\right)\mu \left(x\right)$$

(2) $$\frac{dq\left(t\right)}{dt}=\mu \left(E\left(t\right)\right)$$

To model *B. cereus* growth, the COMBASE model function *μ*(E(t)) is represented by *μ*_max_, the maximum specific growth rate, and this parameter is modelled using a quadratic combination of environmental variables such as pH, temperature (T), and water activity (a_w_). The parameterized model for *μ*_max_ is known as a secondary model in predictive microbiology. 

The function *μ*(E(t)) in COMBASE was derived from a cocktail of *B. cereus* strains, and the parameters from a0 to a9 are linear regression parameters.

(3) $$\begin{array}{ll} \mathrm{In}\left(\mu_{max}\right)=a0+ & a1\cdot T+a2\cdot pH+a3\cdot sqrt\left(1-aw\right)+a4\cdot T\cdot pH+a5\cdot T\\ & \cdot sqrt\left(1-aw\right)+a6\cdot pH\cdot sqrt\left(1-aw\right)+a7\cdot T^{2}+a8\cdot pH^{2}\\ & +a9\cdot \left(1-aw\right) \end{array}$$

Since the linear parameters, a0 to a9, are not available, we have used an estimation procedure to estimate these parameters from a dataset of *B. cereus* growth rates in COMBASE. This dataset was downloaded from the COMBASE data platform (https://browser.combase.cc/, accessed on 15 October 2024), and the SOLVER package from Microsoft EXCEL (see Supplementary Document for details) was used to estimate parameters a0 to a9. The values of these parameter, the SOLVER parameters used, as well as the dataset used, are available in MSEXCEL format upon request from the authors [11].

The alternative source of data used in this study was from the study of Carlin et al. (2013) [8]. The authors used a cardinal-type model to derive the function *μ*(E(t)) for individual *B. cereus* strains. In this cardinal-type framework, the growth rate (*μ*_opt_) is achieved when environmental conditions such as T, pH, and a_w_ are optimal for growth. These optimal conditions are represented by the generic parameter X_opt_. The generic parameter X_min_ represents environmental conditions below which there is no growth, while X*_max_* represents environmental conditions above which there is no growth.

(4) $$\mu_{max}=\mu_{pot}\cdot CM_{2}\left(T\right)\cdot CM_{1}\left(aw\right)$$

(5) $$\begin{array}{l} CM_{n}\left(X\right)\\ =\frac{\left(X-X_{max}\right){\left(X-X_{min}\right)}^{n}}{{\left(X_{opt}-X_{min}\right)}^{n-1}\left[\left(X_{opt}-X_{min}\right)\left(X-X_{opt}\right)-\left(X_{opt}-X_{max}\right)\left(\left(n-1\right)X_{opt}+X_{min}-nX\right)\right]}\\ X\leq X_{min}\\ X_{min}<X<X_{max}\\ X>X_{max} \end{array}$$

In order to generate data from these two sources of information, we had to reconcile the approaches used by COMBASE and Carlin et al. (2013) [8], especially reconciling Equations (3) and (4). We had to reconcile the *μ*_max_ derived from a cocktail of *B. cereus* strains in COMBASE and the *μ*_max_ from individual strains in the Carlin et al. (2013) [8] study [8]. The parameters of the individual strains in the Carlin et al. 2013 study were obtained from the listings in the journal manuscript [8], while the parameters from the COMBASE model were estimated from the dataset available in Table 1.

A practical approach was needed for deriving the open access predictive microbiology *B. cereus* model (open access *B. cereus* model). The approach we chose involves a virtual experiment where the individual strains used in Carlin et al. (2013) are grouped as a cocktail [8]. The Carlin et al. (2013) virtual cocktail growth rate, *μ*_max_, was chosen by applying a MAX function to the range of growth rates of the individual strains calculated from models of the growth for a combination of pH, a_w_, and T using Equation (5) [8]. This was possible since we have the values of the individual strain models which are listed in the journal manuscript. The *B. cereus* cocktail virtual dataset of the Carlin et al. (2013) models will then consist of the data tuple *μ*_max_, pH, T, a_w_, from using the MAX function [8]. Similarly, a data tuple was generated from the COMBASE model, as we have estimates of the parameters of the COMBASE model. The *μ*_max_ from the two sources of data are now reconciled, as both tuples show data from a cocktail of strains. 

The open access *B. cereus* model chosen for this study is based on the approach taken by Ross et al. (2003) [12]. Ross et al. (2003) used a version of the square root model for combining the effects of pH, a_w_, and temperature [12]. This model represents the square root of the maximum specific growth rate (*μ*_max_10) as a function of T, pH, and a_w_. Note that the parameter, *μ*_max_10, is the maximum specific growth derived from bacterial growth curves, where the population is described using logarithm base 10. This parameter, *μ*_max_10, is a transformation of the *μ*_max_ from Equations (3) and (4) which are based on natural logarithms. In the open access model, the environmental values above which the growth rate is zero are represented by the parameters T*_max_* and pH*_max_*, while the parameters T_min_, pH_min_, and a_w(min)_ represent the environmental values below which the growth rate is zero.

(6) $$\begin{array}{ll} \sqrt{\mu_{max10}}=c(T- & T_{min})\left(1-exp\left(d\left(T-T_{max}\right)\right)\right)\cdot \sqrt{aw-aw_{min}}\cdot \sqrt{1-10^{\left(pH_{min}-pH\right)}}\\ & \cdot \sqrt{1-10^{\left(pH-pH_{max}\right)}} \end{array}$$

To estimate the parameters of the open access *B. cereus* model, a virtual dataset from a virtual experiment was generated consisting of 1000 rows of data from random combinations of T, pH, and a_w_. For environmental conditions within the range of the COMBASE model, the data tuple will be based on COMBASE, and for all other environmental conditions, the data tuple will be based on the Carlin et al. 2013 study [8]. These random combinations of T, pH, and a_w_ were chosen from the minimum and maximum ranges of validity of the reconciled models from COMBASE and Carlin et al. (2013) [8]. 

The simulated growth data from the random combination of T, pH, and a_w_ were used to estimate the parameters c, d, T_min_, T*_max_*, pH_min_, pH*_max_*, and a_w_ min of the open access *B. cereus* model (Equation (6)). In the estimation procedure, the initial values used for the parameters were c = 0.1, d = 0.1, T_min_ = 1.4 °C, T*_max_* = 55 °C, pH_min_ = 4.59, pH*_max_* = 8.86, and a_w(min)_ = 0.94. In order to estimate the parameters of the open access *B. cereus* model, a mathematical optimization method was used. The goal of this method is to minimize the difference between the modelled growth rate and the simulated growth data from the virtual dataset.

The Solver function in Excel was the optimization tool used to find the minimum value of a target cell, given certain constraints [13]. The Solver function uses an iterative process to search for the minimum value of the target cell. It starts with an initial set of parameter values and calculates the corresponding modelled growth rate. It then compares the modelled growth rate to the simulated growth data and calculates the sum of the squared errors. The Solver function then adjusts the parameter values and recalculates the modelled growth rate and sum of squared errors. This process is repeated until the sum of the squared errors is minimized.

## 3. Result

**Figure 1:**
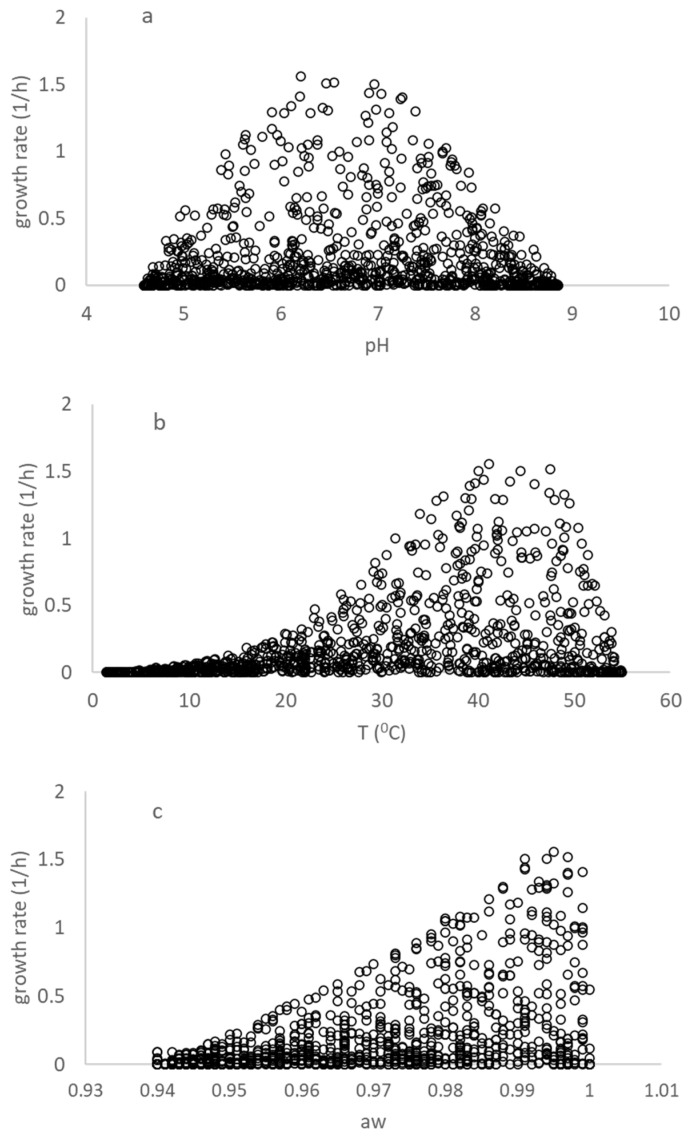
Representation of the growth of a *B. cereus* cocktail originating from the virtual dataset obtained from COMBASE and the study of Carlin et al. (2013) [8]. (**a**–**c**) show the growth rates for a combination of (T, °C), pH and water activity (aw), plotted using the growth rate versus one of individual environmental factor in the virtual dataset.

Figure 1 displays the estimated specific growth rates of the virtual dataset for a selection of temperature (T), pH, and water activity (a_w_). The dataset is comprehensive, containing 1000 rows of data, and covers a range of environmental conditions. The data in Table 2 are a subset of the virtual dataset, and this subset was generated using an algorithm for selecting between the COMBASE and Carlin et al. (2013) models [8]. The choice of using a virtual dataset was a practical and effective solution for reconciling the differences between the growth models used in COMBASE and Carlin et al. (2013) [8], since there are no algebraic solutions for reconciling these models. Therefore, generating this dataset algorithmically was a good solution for use in deriving the adaptive *B. cereus* model (Equation (6)). The use of the virtual dataset and the algorithm for selecting between the models ensures that the fit of the open access model was both comprehensive and accurate.

**Figure 2:**
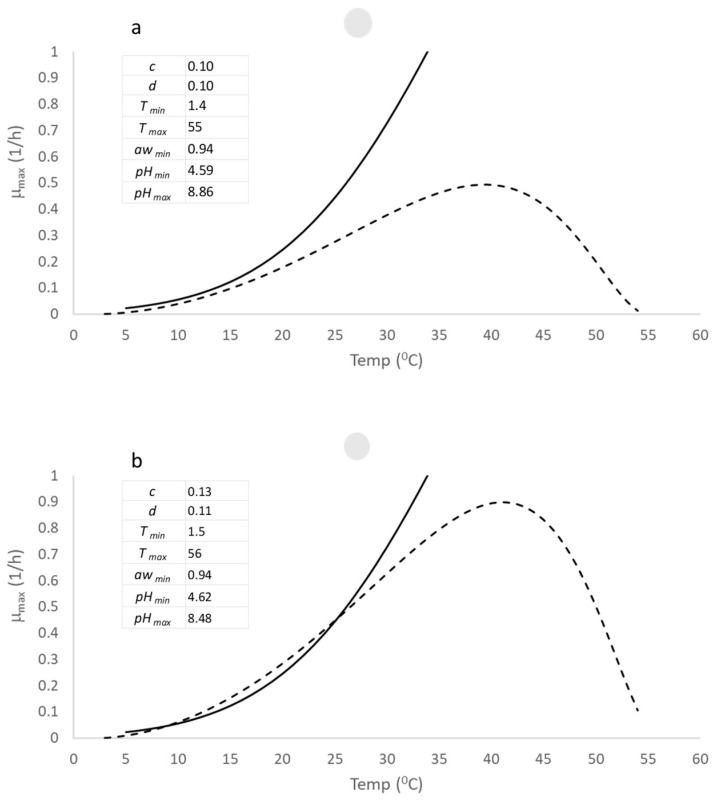
Comparison between the open access *B. cereus* model (-----) and the COMBASE model (–––) at pH 6 and water activity (a_w_) of 0.997 and for the whole growth temperature range. The COMBASE model is only valid in the temperature range from 5–34 °C, and the parameter list of the new model and their values is shown in the upper left-hand corner of the graph. (**a**) shows the comparison using an initial set of parameters and (**b**) shows the results from using the virtual dataset for estimating the parameters using an optimization routine.

Figure 2a provides a comparison between the COMBASE model and an initial iteration of the open access *B. cereus* model using an initial set of parameters (p1, upper left-hand corner). It shows a comparison between the open access *B. cereus* model and the COMBASE model at a pH of 6.7, a water activity of 0.997 and for the complete range of growth temperatures. Note that the validity of the COMBASE model is constrained between 5–34 °C and the deviation between each model arises from the quadratic function used for the COMBASE model. 

A quadratic function is symmetric and will overestimate the growth rate at higher temperatures. The choice of an asymmetric function, given by Equation (6), is therefore reasonable for modelling the whole growth temperature range. The graphs shows that the open access model using p1 had a poor correlation with the COMBASE model. However, after using the virtual dataset and the Solver function in Excel to estimate the updated parameters of the open access model, p2, the correlation improved significantly.

Figure 2b displays the improved correlation between the open access model and the COMBASE model. It demonstrates the effectiveness of the virtual dataset and the Solver function in optimizing the parameters of the open access model. The improved correlation between the open access model and the COMBASE model suggests that the open access model is a good starting point for using these models for the whole range of temperature (T), pH, and water activity (a_w_). 

The upper left-hand corner of Figure 2b displays the estimated parameter set, p2, which is the result of using the virtual dataset and Solver function to optimize the parameters of the open access *B. cereus* model. Since the virtual dataset was generated using random combinations of the minimum and maximum growth limits provided by T_min_ = 1.4 °C, T*_max_* = 55 °C, pH_min_ = 4.59, pH*_max_* = 8.86, and a_wmin_ = 0.94, we would expect the deviations of p2 to be small, given the robust estimation procedure used in this study.

The deviations of p2 from the original parameter values (p1), which are shown in the upper left-hand corner of Figure 2a, are indeed minor, with an order of 5 percent, as the results of the estimation procedure based on 1000 samples show. In summary, the estimation procedure used in this study to optimize the parameters of the open access *B. cereus* model is robust and reliable. The small deviations of the estimated parameter set, p2, from the original parameter values, p1, confirm the effectiveness of the virtual dataset and Solver function in this study. 

Note that by using the MAX function for the Carlin et al. 2013 [8] data, we have effectively removed the growth characteristics of the individual strains of *B. cereus*, e.g., psychotropic or mesophilic modes of growth. We have removed the adaptation of the strains to environmental conditions. The open access *B. cereus* model developed in this study, however, can form a template for validation studies using localized *B. cereus* strains isolated by a food company. 

A set of laboratory experiments from an appropriate experimental design for the localized *B. cereus* strains can then be chosen for estimating or validating the parameters of the open access theoretical *B. cereus* growth models, thereby incorporating the mode of growth, whether psychotropic, mesophilic or thermophilic. The growth data from these laboratory experiments using the strain collection of the food company and the Solver function can then be used to optimize the search for the parameters. This validated model can be used to predict the growth of *B. cereus* for a range of environmental conditions and these personalized *B. cereus* growth models are then tuned to the production facility where the food is produced, thereby allowing the company to identify and mitigate potential risks associated with localized *B. cereus* growth in their products.

One shortcoming using COMBASE, which is free, is the range of applicability. Separation of raw cream in a dairy factory occurs at 50 deg C. If one were to ask how long should the cream separation should last to prevent outgrowth of *B*. *cereus*, then COMBASE predictions are not available. So, industry could try to perform experiments at 50 deg C, in order to answer this question. In addition, heating and cooling times of food products many contain ranges which are not available in COMBASE. We published this open access model as a solution to these questions, which are routine in the food industry. This open access model is an attempt to make it easier for industry to implement prediction microbiology themselves. However, there are proprietary software platforms, but these platforms charge users for their use.

Also, once these parameters are validated, the relevance of using this model in a risk assessment of the company’s products becomes more structured. The use of a validated predictive model will enable the company to take informed decisions about food safety and identify potential risks before they become a problem. The open access model can also be used to convince regulators of the enhanced safety assessment of the company’s products and provide a more rigorous approach to regulatory compliance.

The personalized *B. cereus* growth models can also be used to support the development of effective intervention strategies for controlling *B. cereus* growth in unique food production environments. By incorporating strain-specific data from production facilities, the model can be tailored to the unique conditions of individual manufacturing sites. This customization enables more targeted and efficient control measures, ultimately reducing the risk of *B. cereus* contamination in food products [14].

Future research could focus on further refining and validating the open access *B. cereus* model, incorporating additional environmental factors and other pathogen strains to enhance its versatility and applicability across a wider range of food products. As more data become available, the model can be continuously updated and improved, ensuring that it remains a valuable tool for the food industry.

In addition to refining the model itself, future research could explore novel ways to integrate the open access *B. cereus* model into decision support tools and software platforms for the food industry. By developing user-friendly interfaces and incorporating advanced data visualization techniques, researchers can help make the model more accessible and useful for food safety professionals. 

Furthermore, future research could explore the potential applications of the open access *B. cereus* model in other food industries, such as meat and poultry, or produce.

## 4. Conclusions

In summary, the open access *B. cereus* model, parameterized by c = 0.13, d = 0.11, T_min_ = 1.5 °C, T*_max_* = 56 °C, pH_min_ = 4.62, pH*_max_* = 8.48, and a_wmin_ = 0.94, is a useful tool for food companies in assessing the safety of their products. A company could generate a set of growth data using a company-localized strain cocktail and use these localized data to adapt the parameters of the generalized open access model, thereby providing a customized and accurate predictive microbiology model for the specific products of the company. 

The use of a validated, personalized predictive microbiology growth model will enable the company to take informed decisions about food safety and comply with regulatory requirements. The generalized open access model has the potential to revolutionize the way companies assess the safety of their products and ensure that consumers are protected from potential health risks associated with *B. cereus* growth in food.

## Figures and Tables

**Table 1:** A subset of the *Bacillus cereus* growth rate, downloaded from the COMBASE platform (https://browser.combase.cc/, accessed on 15 October 2024) on 6 May 2024. The environmental conditions were chosen at random from the domain of validity of the COMBASE *B. cereus* growth model, which includes pH ranges from 4.9 to 7.4, temperature ranges from 5 to 34 °C, and water activities from 0.94 to 1.

T (°C)	pH	a_w_	Growth Rate (1/h)
5	4.9	0.94	0.002
25.6	6.6	1	0.516
16.1	6	0.97	0.053
5.4	5.7	1	0.017
25.1	6.7	0.98	0.314
18.6	7.2	0.97	0.125
19.5	6.4	0.95	0.039
20.6	6.1	0.99	0.212
5.6	6.6	0.98	0.019
13	5.8	0.97	0.032

**Table 2:** A subset of the virtual dataset ^(1)^ of size n. The combinations of pH, temperature (T, °C) and water activity (a_w_) were randomly selected from a uniform distribution of temperature T (1.4–55 °C), pH (4.59–8.86) and a_w_ (0.94–1.00), and these were used to calculate the growth rate (1/h). *n* = 1000.

Growth Rate (1/h)	T (°C)	pH	a_w_	Source
0	1.4	4.59	0.94	b
0.07	23.2	4.93	0.97	a
0.15	20.3	6.79	0.97	a
0.07	26.5	5.63	0.96	a
0.04	13.2	7.32	0.96	a
0.17	21.1	6.64	0.98	a
0.59	28.8	5.96	0.99	a
0.55	28.7	8.03	1.00	b
0.02	15.2	6.69	0.95	a
0.03	10.5	7.33	0.97	a
1.13	43.2	6.27	0.98	b
0.38	48.6	5.14	0.99	b
0.06	11.9	6.08	0.98	a
0.19	33.2	5.20	0.98	a
0.01	5.3	7.28	0.94	a
0.26	37.4	8.54	0.99	b

^(1)^ For environmental conditions within the range of COMBASE model (a), the data tuple will be based on COMBASE, and for all other environmental conditions, the data tuple will be based on the Carlin et al. 2013 [8] study (b).

## Data Availability

The original contributions presented in the study are included in the article/supplementary material, further inquiries can be directed to the corresponding author.

## Supplementary Materials

The following supporting information can be downloaded at: https://www.mdpi.com/article/10.3390/foods13213382/s1, Document for details.
